# Visualizing sound: counting wolves by using a spectral view of the chorus howling

**DOI:** 10.1186/s12983-015-0114-0

**Published:** 2015-09-15

**Authors:** Daniela Passilongo, Luca Mattioli, Elena Bassi, László Szabó, Marco Apollonio

**Affiliations:** Department of Science for Nature and Environmental Resources, University of Sassari, via Muroni 25, I-07100 Sassari, Italy; Servizio Piano Faunistico, Provincia di Arezzo, Piazza della Libertà 3, I–57100 Arezzo, Italy; Szent István University, Institute for Wildlife Conservation, Páter K str 1, Gödöllő, Hungary

## Abstract

**Introduction:**

Monitoring large carnivores is a central issue in conservation biology. The wolf (*Canis lupus*) is the most studied large carnivore in the world. After a massive decline and several local extinctions, mostly due to direct persecutions, wolves are now recolonizing many areas of their historical natural range. One of the main monitoring techniques is the howling survey, which is based on the wolves’ tendency to use vocalisations to mark territory ownership in response to howls of unknown individuals. In most cases wolf howling sessions are useful for the localisation of the pack, but they provide only an aural estimation of the chorus size.

We tested and present a new bioacoustic approach to estimate chorus size by recording wolves’ replies and visualising choruses through spectrograms and spectral envelopes. To test the methodology, we compared: a) the values detected by visual inspections with the true chorus size to test for accuracy; b) the bioacoustic estimations of a sample of free-ranging wolves’ replies developed by different operators to test for precision of the method; c) the aural field estimation of chorus size of a sample of free-ranging wolves’ replies with the sonogram analysis of the same recordings to test for difference between methods.

**Results:**

Visual inspection of the chorus by spectrogram and spectrum proved to be useful in determining the number of concurrent voices in a wolf chorus. Estimations of chorus size were highly correlated with the number of wolves counted in a pack, and 92 % of 29 known chorus sizes were recognized by means of bioacoustic analysis. On the basis of spectrographic evidence, it was also possible to identify up to seven concurrent vocalisations in a chorus of nine wolves. Spectral analysis of 37 free ranging wolves’ replies showed a high correlation between the chorus size estimations of the different operators (92.8 %), but a low correlation with the aural estimation (59.2 %).

**Conclusions:**

Wolf howling monitoring technique could be improved by recording wolves’ replies and by using bioacoustic tools such as spectrograms and spectral envelopes to determine the size of the wolf chorus. Compared with other monitoring techniques (i.e., genetic analysis), bioacoustic analysis requires widely available informatic tools (i.e., sound recording set of devices and sound analysis software) and a low budget. Information obtained by means of chorus analysis can also be combined with that provided by other techniques.

Moreover, howls can be recorded and stored in audio file format with a good resolution (i.e. in “Wave” format), thus representing a useful tool for future listening and investigations, which can be countlessly employed without risks of time deterioration.

**Electronic supplementary material:**

The online version of this article (doi:10.1186/s12983-015-0114-0) contains supplementary material, which is available to authorized users.

## Introduction

Monitoring and managing the recovery of large wide-ranging mammalian carnivores are major issues for both conservation biologists and wildlife managers [1]. In the light of the continuous habitat loss and direct persecution of these species, large carnivores conservation has become a pressing need [2]. Often described as “charismatic species” [3], these carnivores actually have a high ecological [2, 4], economic and social impact [3, 5–7] and, therefore, an in-depth knowledge of their ecology and behaviour is highly desirable for the development of an effective conservation strategy.

At the beginning of the 1990s, after a massive decline caused by centuries of direct persecution, deforestation and overhunting of its natural prey [8, 9], the wolf (*Canis lupus*), became almost extinct in Western Europe, United States and Mexico [4]. At the present, thanks to legal protection and socio-economic changes in Europe, as the improvement in habitat quality and the presence of large populations of wild ungulates [10]; the relocation effort [11] and areas with vast public lands [12] in US, wolves have reoccupied 67 % of their historical range worldwide [4]; in this context, monitoring wolf population is a crucial issue for wolf conservation efforts.

One of the main wolf monitoring tools is the howling survey [13]: given the tendency of resident wolves to respond to extraneous vocal stimuli in order to defend the resources in their territories and to avoid encounter with neighbour packs, this approach consists in the acoustic stimulation produced through human simulation or playback of actual wolf howls [14–16]. This method was employed in several studies for monitoring and censusing wolf packs [15, 17–19] and other species with similar vocal behaviour [20, 21]. However, in most cases, successful field wolf howling sessions end with the pack localisation and an aural estimation of chorus size, and only few attempts to determine chorus size through complex sound and statistical analysis have been made [22–26]. In this regard, several studies have already found both individual [26–28] and group vocal signature [29] by using spectrographic analysis, thus emphasising the high potential of bioacoustic tools to improve the knowledge on this species. Nevertheless, no study has yet used available bioacoustic softwares and visual spectrographic inspection of the howling to estimate chorus size.

In most cases chorus size is estimated aurally, by counting each individual as it joins the chorus [15, 30]. However, precise estimation of the number of wolves is difficult with this method because only the first two or three wolves enter in the chorus as a staggered basis followed by the rest of the pack en masse [15]. Moreover, the packs often reply before the end of the stimulus, and, in particular, packs with more adults tend to reply more quickly [15, 22, 30]. Additionally, since subordinate adults’ and pups’ howls consist in a rapid frequency modulation, they add complexity to the chorus and even experts may encounter difficulties in counting them (see [15]). Counting ability in humans has been tested also in the case of human voices [31] and music instruments [32]. In both cases, human aural perception generally failed to count more than three concurrent sources, however, during the test on the denumerability of music instruments, musicians performed about 20 % better than non-musicians [32], thus highlighting the variability level associated to individual background and expertise. Consistent with these findings, studies on the aural denumerability of chorus size also recognised about 3 wolves [15, 22].

For this reason, researchers conservatively set a fixed pack size to their data collected through howling survey (see [33, 34]), and, if possible, determine the pack size and the aggregation rate of the packs of their target populations using other, often more expensive and time-consuming techniques (genetic: [34, 35], tracks: [33], VHF/GPS collars: [36, 37]; camera traps: [38]). Since during the reply some individuals of the pack could stay silent [16] or be temporarily absent [37], chorus size does not necessarily correspond to the actual pack size; even so, chorus size can be used as a proxy of the minimum pack size. As for wolves, the pack is the basic social unit [39] and pack size is correlated with several ecological traits such as hunting efficiency and prey selection [40, 41], pack size estimation is a key-issue for wolf monitoring, research, and conservation purposes.

In this paper, we present a bioacoustic approach based on the visual inspection of the sound to estimate chorus size of the pack. We validated the method by evaluating both its accuracy (comparison of the chorus size estimated through the method with the real chorus size) and its precision (comparison of the chorus size estimated through the method by two operators) The efficiency of the method was then proved by comparing the chorus size estimated through visual inspections with the estimation of the chorus size based on an aural estimation.

Therefore, the aim of this study was to test the benefits of using bioacoustic analysis to estimate wolf chorus size by recording and analysing wolves’ choruses during the wolf howling monitoring sessions, thus highlighting a further connection between behavioural and conservation issues.

## Results and discussion

### Accuracy of the method

**Fig. 1:**
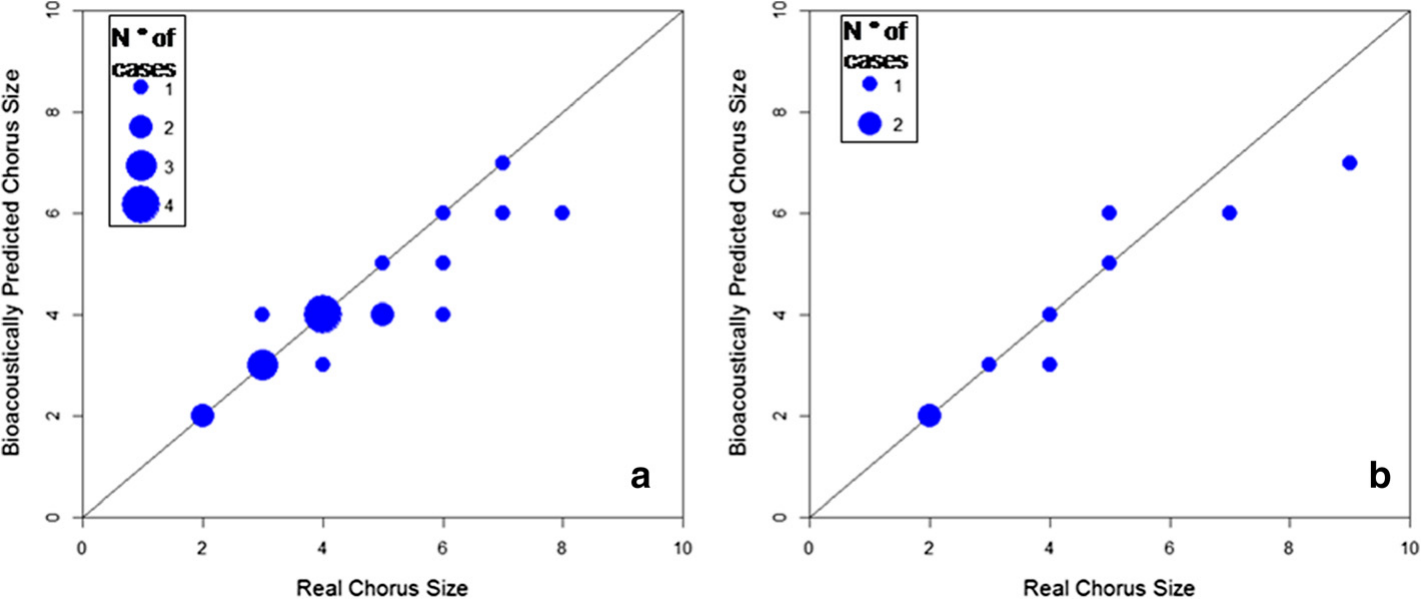
Bioacoustically predicted versus Real chorus size. Scatter plots representing real versus bioacoustically predicted chorus size for Human Simulated (HSH) (panel **a**) and Wolf Downloaded Howling (WDH) (panel **b**) tests. Radiuses are proportional to the number of cases. Estimation was exact in 12 cases out of 20 for HSH and 5 cases out of 9. The diagonal represents the 1:1 correlation

**Fig. 2:**
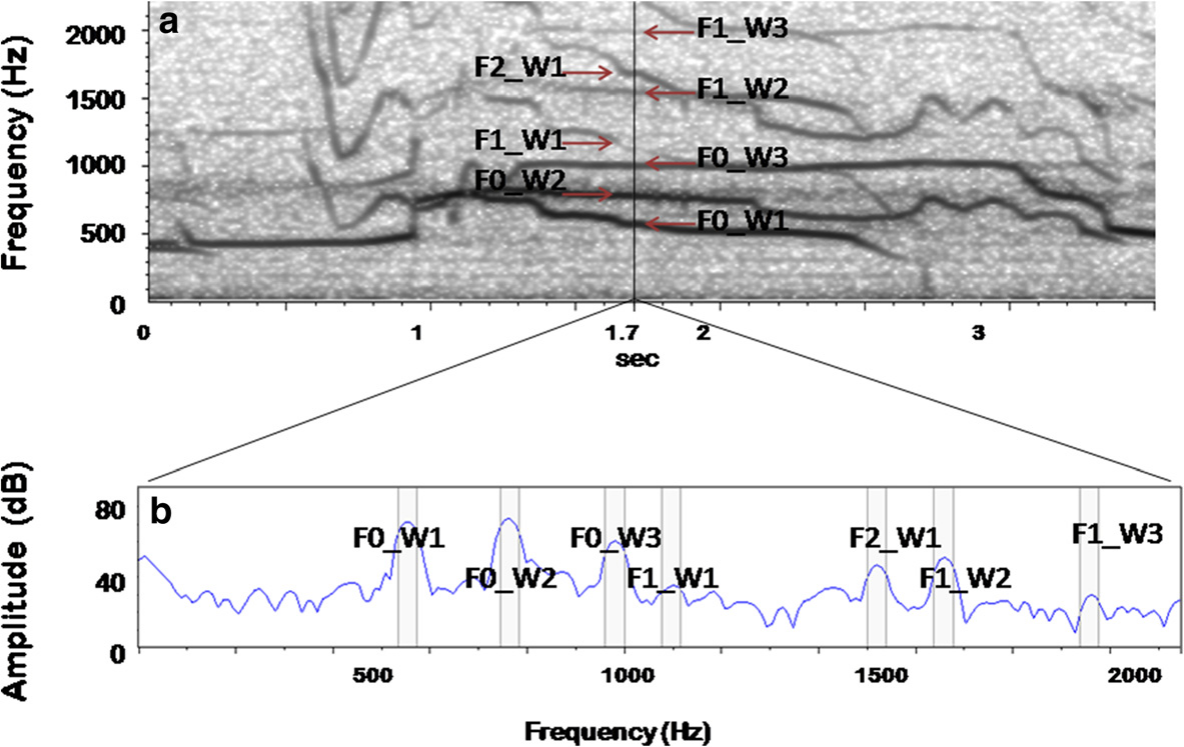
Spectral components of a wolf chorus. Narrow band spectrogram (DFT size: 2048 samples; Hanning window; frequency grid: 21.5 Hz; time step: 10 ms; bandwidth: 37.5 Hz) showing 3.5 s of a wolf chorus emitted by a free ranging pack and recorded during the howling survey. Spectrogram (**a**) and its spectral envelope (**b**) at the second 1.7 are presented. Three different howls recognizable because of the different shape of F0 and harmonic structure, are present at the same time. Other amplitude picks are due to background noise and echoes. Legend: W = wolf ; F0 = fundamental frequency; F1 = first harmonic; F2 = second harmonic

Visual estimation by spectrogram and spectrum provided an accurate evaluation of the chorus size as a strong positive correlation was found between real and estimated values in both our tests of known chorus size (Human simulated howling, HSH: *n* = 20, Spearman’s rho = 0.90, *p* < 0.0001; and Wolf downloaded howling, WDH: *n* = 9, Spearman’s rho = 0.97, *p* < 0.0001), (Fig. 1) with no difference between real and estimated means (Wilcoxon signed rank test, HSH: *n* = 20, *Z* = 2.12, *p* = 0.057; WDH: *n* = 9, *Z* = 1.13, *p* = 0.501). The most common error was the underestimation (false negative) for one individual (five and two cases in HSH and WDH), followed by the underestimation of two individuals (two and one cases) (Fig. 1). Overestimation (false positive) occurred twice, with four concurrent voices estimated in a chorus size of three (in HSH) and six concurrent calls estimated in a chorus of five individuals (in WDH) (Fig. 1). Choruses composed by two individuals were always correctly estimated and it was even possible to recognise up to six different voices in a chorus of eight contemporaneous voices (in HSH test) and up to seven different voices in a chorus of nine elements (in WDH test). In the two cases of overestimation, the detection of the “phantom voice” was probably due to the misinterpretation of a mix of non-linear (deterministic chaos, subharmonics) [42, 43] and environmental phenomena (reverberation, echoes) [44]. In fact, while harmonic overtones were easily recognised because of their shape (the same as F0) and frequencies (integer multiple of F0) (Fig. 2) and, therefore, hardly misinterpreted as a different fundamental frequency (one more individual), other phenomena could affect and limit chorus visualisation. However, repeated measures in different parts of the chorus could effectively limit overestimation errors.

Considering the overall sample (test HSH and WDH), 92 % of the total number of voices were recognised by the visual analysis of the choruses, thus highlighting the potential and wide applicability of this methodology.

**Fig. 3:**
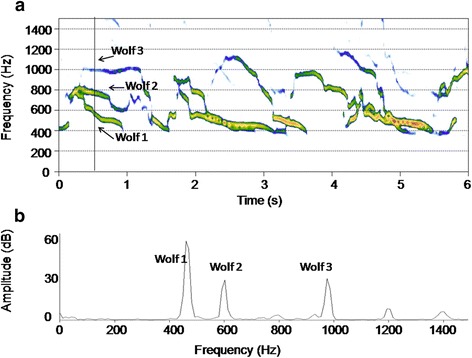
Three wolves’ choral howls. Chorus howls of at least three different wolves (free-ranging) recorded during the howling survey in 2007. Spectrogram (**a**) and spectrum (**b**) (window length: 4026 samples) were computed by Seewave, an *open source* R project package dedicated to the sound analysis. Colours (*from red to blue*) represent amplitude degradation. See also Additional file 1 and Additional file 2 for analysis of wolves choruses with Seewave

In the last few years, like all the inherent computer science technologies, analysis tools such as spectrograms and spectral envelopes based on the digital Fourier transform [45] have become accessible to a broad range of researchers [46]. This was possible thanks to the spread of several highly interactive software (e.g., Raven, made available by Cornell University [47], and widely accessed *open source* software environments and programming languages such as R [48], with its Seewave package specifically dedicated to time series (i.e. sounds) visualisation and analysis [49] (see Fig. 3 and Additional file 1 and Additional file 2 for an example of spectrographic and spectral visualisation of a wolves chorus).

### Precision of the method

Results from the visual estimation performed by another independent operator after the training on the methodology, also showed high correlation values for both test (Human simulated howling, HSH: *n* = 20, Spearman’s rho = 0.92, *p* < 0.0001; and Wolf downloaded howling, WDH: *n* = 9, Spearman’s rho = 0.80, *p* = 0.009); and no statistical difference between real and estimated means (Wilcoxon signed rank test, HSH: *n* = 20, *Z* = 1.73, *p* = 0.147; WDH: *n* = 9, *Z* = 0.74, *p* = 0.495) confirming the accuracy and showing the precision of the bioacoustic method.

**Fig. 4:**
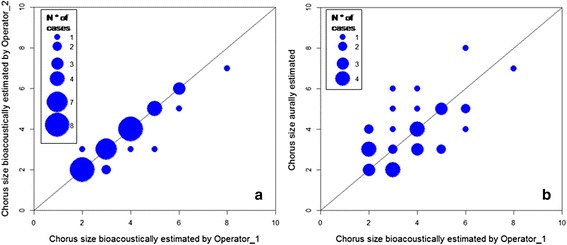
Comparison between estimated chorus size bioacoustically predicted from two different operators and between aural and bioacoustic. Scatter plots showing comparison between bioacoustic estimation performed by two operators and aural and bioacoustics estimations of 37 chorus. Bioacoustic estimations by visual inspection of independent operators (Panel **a**) were highly correlated. Aural and bioacoustic estimations (Panel **b**) were poorly concordant. Radiuses are proportional to the number of cases. The diagonal represents the 1:1 correlation

Similarly, analysis of free ranging wolves’ replies showed a high correlation between bioacoustic estimations of chorus sizes by independent operators (Spearman’s rho = 0.93, *p* < 0.0001) with no difference between operators’ means (Wilcoxon, *n* = 37, *Z* = −1.898, *p* = 0.112). Chorus size estimation by visual inspection was the same for both operators in 30 replies out of 37 (88 %), and operator 2 agreed with operator 1 in 131 out of 138 total voices classification. Maximum difference between operators was of two individuals in only one case and one individual in six cases (Fig. 4a), thus showing a high precision of spectrogram analysis.

### Efficiency of the method

Comparison of aural estimations of the 37 free ranging wolves’ replies with the bioacoustic estimations (Fig. 4b) showed low correlation in both cases (vs. Operator 1: Spearman’s rho = 0.59, *p* = 0.0001; and vs. Operator 2: rho = 0.63, *p* < 0.0001), which thus proved the two methods do not provide the exact same results.

Aural and bioacoustic estimations showed similar overall mean pack size, i.e. comparable accuracy, but very low concordance that is different precision. Mean chorus size obtained from bioacoustic and aural method did not differ statistically (Wilcoxon, *n* = 37, Operator 1 vs. Aural: *Z* = 0.446; *p* = 0.689; Operator 2 vs. Aural: *Z* = 1.362; *p* = 0.186) as a consequence of bi-directional differences between aural and bioacoustic estimations. Aural and visual pack size estimations were identical in only 12 cases (32 %); a difference of one individual was found in 16 cases, a difference of two individuals was found in eight cases. Finally, three more individuals were estimated aurally with respect to the bioacoustic estimation in one case (Fig. 4b).

For polyphonic sounds, visualisation is a source separation method [46] and, below certain sizes, good spectrographic exams of wolf choruses can realistically reveal a number of different contemporaneous sources. However, as the number of contemporaneous sources increases (7–9 sources, depending on the quality of the recordings and the chorus modulation), the overlap between howls generates a high-density spectrogram (and spectral envelope), in which the different components of the sound can hardly be discriminated.

Here we showed a high correlation between bioacoustic estimation performed by visual inspection of the chorus and real chorus size and between the estimations of different operators, thus highlighting the precision of the technique. Sound editing can also improve listening quality: indeed, most of the factors affecting aural field estimation (high background noise) can be totally (chickadees) or partially (in case of overlap with the target frequencies - e.g., airplanes’ engines) removed (filtered out) during the sound analysis phase.

Conversely, we showed.discrepancies between aural and bioacoustic chorus size estimations. Previous works [15, 22] also showed that aural field estimation of wolf packs commonly failed to count more than three wolves correctly, consistent with human general failure to recognise more than three concurrent sources in a polyphonic contest, both in the case of human voice [31] and music instruments [32].

Although chorus size does not necessarily correspond to pack size [16, 37] and further studies are needed in order to better determine the age class of the members and the overall active space of the choruses, we believe that this method represents a very useful tool and a relative low-budget technique for the estimation of minimum wolf pack size.

## Conclusions

In this paper, we presented a bioacoustic approach based on visual spectrographic inspections of wolves’ howling to handily estimate the chorus size of the pack. We demonstrated that the process of recording and analysing wolves’ replies collected during the howling survey has several advantages. Once recorded, a sound is available for a potentially infinite number of listening sessions. Moreover, in order to improve the quality of the sound and, consequently, of the chorus size estimation, these recordings can also be edited (i.e., filtered, amplified) and saved in modified versions. For this reason, we suggest to save the original files for possible future studies. Also, prerequisite for a good spectrographic analysis and thus for a good estimation of the chorus size is the recording quality: an ultra-directional microphone, uncompressed “wave” or “aif” format and an adequate sampling frequency ( 22,000 or 44,000 Hz) are essential characteristics for recording the howls correctly.

Recordings of wolves’ replies and bioacoustic analysis can help determine chorus size, thus allowing to count different wolves in a chorus in a more precise and less sensitive way than with the more subjective, extemporaneous field estimation by ear.

However, in order to be effectively used, the application of the methodology for regular monitoring of wolf populations requires a preliminary training and, for this reason, researchers will have to instruct and guide managers in the use of the technique described. A bioacoustic approach to the howling survey can help wildlife and conservation biologists monitor wolves as well as other canid species with similar acoustic territorial marking behaviour, such as the coyote (*Canis latrans*), and the golden jackal (*Canis aureus*), offering a realistic, objective (because based on the spectral components of the choruses) estimation of any pack size of up to six-eight members and thus, the possibility to track this trend.

Since this source of information can be combined with other sources (e.g., genetic samples [50], snow tracks [34], remote photo-videos [51]) in a cross-modal monitoring, we believe that chorus size estimations by means of bioacoustic analysis can help biologists monitor wild populations of vocal animals, through non-invasive methods and properly inform administrators on the conservation strategies required, on the basis of a realistic estimate of the status and trends of these predators.

## Materials and methods

### Tests procedure

We evaluated the reliability of the chorus size estimation by visual inspection of spectrogram and spectrum. First, we measured the accuracy of the estimated chorus size. During a howling survey there is generally no visual access to the replying pack. We thus analysed bioacoustically choruses of known size, either simulated by humans (i.e. “Human simulated howling” test, HSH; *n* = 20), or of real wolves (i.e. “Wolf downloaded howling” tests, WDH; *n* = 9). Second, we estimated the precision of the technique by comparing bioacoustically estimated values of 37 free-ranging wolves’ choral replies from two independent operators. A training on bioacoustic analysis following the procedure highlighted in this paper was performed by operators before the tests.

Finally, we tested whether aural estimations and estimations from visual inspection of spectrogram and spectrum provided comparable results. Using the same 37 wolves’ replies we compared aural chorus size estimations obtained in the field during the howling survey (not necessarily by the same field operators) with the bioacoustics estimations of the same choruses.

### Data acquisition

Human-simulated howls (HSH) were recorded in summer 2012 and 2013, by groups of 2 to 8 volunteers who were asked to howl together after training on howling simulation. Breaking and flat howls were alternated, with at least 5 howls per trial, and one individual entering in the chorus as a staggered basis, following Harrington and Mech [14]. Human howls were recorded in Fonte del Baregno (43°62’ N, 11°93’ E), within the protected area called Alpe di Catenaia in the Apennine Mountains, in the North-East of Tuscany, Italy. Distance between source and recorder was 100 m.

Choruses from the internet (Wolf Downloaded Howls, WDH) were downloaded from YouTube as video file (.flv) format with VSO downloader 2.9.12 [52] after a research with keywords such as “wolf”, “howls” or similar terms. We selected and downloaded 9 videos in which it was effectively possible to count the howling wolves and all the howling wolves were well visible. So as to be sure that the chorus size corresponded to the pack size, 8 out of the 9 choruses used in the WDH were recorded in captivity (especially in the zoo). The videos were then converted from the original video format (MP4, “.mov” or “.flv” types) into audio format (2 channels, Wave format, 44,100 KHz and 16 bit format) with the software 4Free Video Converter [53] . All the links to the original files are in Additional file 3.

Free-ranging wolves’ replies were collected from 2008 to 2014 during a wolf howling monitoring program (following the Habitat Directive on priority species [92/43/EEC]) carried out in the Province of Arezzo (3230 km2), Eastern Tuscany, Italy.

Wolf howling survey was performed in summer (from July to October), when the pack activity was focused in the home-sites, because of the pups presence and the rate of response was consequently higher [14, 15, 54]. Sampling sites were chosen so as to cover the whole study area, following the method described by Harrington and Mech [15] as “saturation census” and adapting it to local requirements/topography to maximise the range of audibility and minimise sound dispersion [54]. Following the standard procedure suggested by Harrington and Mech [15]: i) no session was conducted during rainfalls nor with strong wind; ii) wolf howling was performed overnight, to minimise the anthropogenic noise; iii) two trials were conducted per site.

Wolves respond to the howling of unfamiliar individuals in six different basic ways, from retreating silently to remaining and replying with/by vocal approach [14] in relation to their resources (e.g. fresh prey), and social context (e.g. presence of pups) and to the stimulus [16]; moreover, they respond to human simulated howling as well as to playbacks [13, 14]. For these reasons, our stimuli always consisted in a chorus howls emitted by two individuals (howling playback by a captive pair of wolves (duration: 1.20 min) or by human simulated howling (duration: *circa* 1 min)). Playback of recorded chorus howls was emitted by an exponential horn with high emission directionality (120° horizontal coverage and 60° vertical).

Three minutes after the first stimulus, if no answer had followed, a second trial (higher in volume to cover a bigger area) was attempted, after which the operators left the site. In case of response, reply bearing, times and an extemporaneous estimation of the chorus size by ear (from the operator without headphone and microphone) were recorded for each answer. For a better localization of the pack we repeated one or more trials from a place closer to the presumed site of response or concurrent sessions were performed by two groups of operators. Real pack sizes were unknown for the free-ranging wolves.

Humans’ and free-ranging wolves’ howls were captured with a Sennheiser directional microphone fitted with a windshield (ME67 head with K6 power module – frequency response: 50–20,000 Hz) and saved on a hand-held M-Audio Microtrack 24/96 II digital recorder, in uncompressed Wave format with a 44,100 Hz sampling rate and 16 bits amplitude resolution.

### Bioacoustic analysis

Acoustic signals were analysed with Raven pro 1.4, developed by Cornel Lab of Ornithology) [47], and with the *open source* Seewave package [49] in R v. 2.9.0 [48] to implement the spectral view.

To estimate chorus size by visual inspections, spectrograms and spectral envelopes were computed for each audio file (Figs. 2 and 3). Spectral envelope (or *spectrum*) represents the sound at a given instant, showing the frequencies on the horizontal axis and the sound pressure (or amplitude) on the vertical axis [47, 55, 56]. The spectrogram of a sound represents instead a sequence of spectra, showing time on the horizontal axis, frequency on the vertical axis, and the sound pressure as a greyscale or different colour scale (Fig. 3) [47, 55, 56]. Spectrogram and spectrum are based on the mathematical function Fourier transform [47, 55, 56], and the version of this function which is used to represent digitalised/discrete signals is called discrete Fourier transform (DFT) [47]. DFT size represents the length of the analysis window (the window size), and thus the number of frames sampled to compute each spectrum of the spectrogram, while the window function (i.e., Hanning, Gaussian) determines how to taper the abruptness of the onset and offset of a segment [56]. A narrow-band spectrogram (high window size values) results in a spectrogram with frequencies which clearly differ from one another. To analyse wolves choruses, parameters were set as follows: DFT size: 2048 samples; Hanning window; frequency grid: 21.5 Hz; time step: 10 ms, where frequency grid = (sampling frequency)/DFT size, while time step was taken to be the distance between the centre of subsequent samples. In case of noise in the recordings (anthropogenic: cars, planes, high music from villages, human voices, bells; natural: wind, rivers, other animals), a band-pass flat filter (100–2000 Hz) was applied to delete noise and thus to improve the audibility of wolves’ replies.

Every single howl emitted by a wolf appears as a fundamental frequency (F0) and its harmonic overtones, or harmonics (Fig. 2). The fundamental frequency is the glottal pulse rate and determines the pitch of the voice [56], while harmonic overtones are integer multiples of the fundamental frequency (F0*2; F0*3;…, F0*N) [56].

Chorus size was then estimated by counting the number of different howls (viewed as the fundamental plus harmonics) visualised at the same time (Fig. 2)), assuming that one wolf cannot produce multiple fundamental frequencies at a given time. Harmonic overtones were easily recognised because of their shape (same as F0) and frequencies (integer multiples of F0); since the difference between howls (inter-harmonic space) doubles from the fundamental frequencies to the first harmonics, they can also help recognise different howls (Fig. 2).

### Statistical analysis

All statistical analyses were carried out using R v. 2.9.0 [48]. Spearman’s rank correlations were computed to compare the real and bioacoustically predicted chorus size, the bioacoustic estimations of the chorus sizes performed by two different operators and the bioacoustic and aural estimation of the chorus size. Hypotheses of no mean differences between the real and bioacoustically predicted chorus size, the bioacoustic estimations of the chorus sizes performed by two different operators and the bioacoustic and aural estimation of the chorus size were tested by Wilcoxon matched-pairs signed-ranks test.

## Additional files

Additional file 1:
**R Script for wolf choruses analysis with Seewave package in R environment.** (R 1 kb) Additional file 2:
**Audio file of free-ranging wolf howling.** (WAV 7962 kb) Additional file 3:
**Links to the videos used for WDH test.** (DOCX 12 kb)
