# Supplementary material for: Visualizing sound: counting wolves by using a spectral view of the chorus howling
Source: Front Zool. 2015 Sep 15;12:22. doi: 10.1186/s12983-015-0114-0 (PMC4570177; doi:10.1186/s12983-015-0114-0)
Supplement: Additional file 3: — Links to the videos used for WDH test. (DOCX 12 kb) [file 12983_2015_114_MOESM3_ESM.docx]

**Additional file 3: Link to the files**

Video files used for WDH test, to determinate the efficiency of the pack size estimation.

| **Title** | **N° of Wolves** | **Link** |
| --- | --- | --- |
| 4-5 weeks old howling wolves | 4 | <https://www.youtube.com/watch?v=ZWNMqkiQ5tM> |
| 4 months pups howling | 5 | https://www.youtube.com/watch?v=p33VyO8s9o8 |
| Young wolves howling | 3 | <https://www.youtube.com/watch?v=JD3xEcAr3Ks> |
| Wolf howl in Asahiyama | 2 | <https://www.youtube.com/watch?v=tupOeH4bcfw> |
| Wolves Howling | 3 | <https://www.youtube.com/watch?v=V3qA5eZwywA> |
| Grey Wolves Howling | 4 | <https://www.youtube.com/watch?v=iWbKlbcEQrI> |
| Chorus howling in captivity | 9 | <https://www.youtube.com/watch?v=mgdC2zZoGlE> |
| Howling Wolves medium | 7 | http://youtu.be/hYKY6GW6ENc |
| Pack Rally and the Start of Testing Behavior | 5 | <https://www.youtube.com/watch?v=zW7GZws661k&list=PL96F380E390ECC5E1> |
